# Strategies for Surface Modification with Ag-Shaped Nanoparticles: Electrocatalytic Enhancement of Screen-Printed Electrodes for the Detection of Heavy Metals

**DOI:** 10.3390/s19194249

**Published:** 2019-09-30

**Authors:** Karina Torres-Rivero, Lourdes Torralba-Cadena, Alexandra Espriu-Gascon, Ignasi Casas, Julio Bastos-Arrieta, Antonio Florido

**Affiliations:** 1 Departament d’Enginyeria Química, Escola d’Enginyeria de Barcelona Est (EEBE), Universitat Politècnica de Catalunya, BarcelonaTEch (UPC), Av. Eduard Maristany 16, 08019 Barcelona, Spain; ltc_86@hotmail.com (L.T.-C.); alexandra.espriu@upc.edu (A.E.-G.); ignasi.casas@upc.edu (I.C.); antonio.florido@upc.edu (A.F.); 2 Barcelona Research Center for Multiscale Science and Engineering, Av. Eduard Maristany 16, 08019 Barcelona, Spain; 3 Physical Chemistry TU Dresden, Zellescher Weg 19, 01069 Dresden, Germany

**Keywords:** screen-printed electrodes, Ag nanoparticles, drop-casting, spin-coating, nanoprisms, heavy metals, differential pulse anodic stripping voltammetric, electrocatalysis

## Abstract

Screen-printed carbon nanofiber electrodes (SPCNFEs) represent an alternative with great acceptance due to their results, as well as their low impact on the environment. In order to improve their performance, in the present work they were modified with silver nanoparticles (Ag-NPs) and electrochemically characterized by using anodic stripping voltammetry. From the Ag-NP synthesis, silver seeds (Ag-NS) and silver nanoprisms (Ag-NPr) were obtained. The Ag-NP formation was confirmed by micrographs, where Ag-NPs with diameters of 12.20 ± 0.04 nm for Ag-NS and 20.40 ± 0.09 nm for Ag-NPr were observed. The electrodes were modified by using three different deposition methods—drop-casting, spin-coating, and in situ approaches—that offer different nanoparticle distribution and electrode modification times. It was observed that the last methodology showed a low amount of Ag-NS deposited on the electrode surface and deep alteration of this surface. Those facts suggest that the in situ synthesis methodology was not appropriate for the determination of heavy metals, and it was discarded. The incorporation of the nanoparticles by spin-coating and drop-casting strategies showed different spatial distribution on the electrode surface, as proved by scanning electron microscopy. The electrodes modified by these strategies were evaluated for the cadmium(II) and lead(II) detection using differential pulse anodic stripping voltammetry, obtaining detection limit values of 2.1 and 2.8 µg·L^−1^, respectively. The overall results showed that the incorporation route does not directly change the electrocatalytic effect of the nanoparticles, but the shape of these nanoparticles (spherical for seeds and triangular for prisms) has preferential electrocatalytic enhancement over Cd(II) or Pb(II).

## 1. Introduction

In order to ensure water quality, the World Health Organization (WHO) has published the maximum allowed concentration of pollutants in drinking water [1]. Among these pollutants, the concentration of heavy metal ions (HMI) is of special concern, due to their toxicity and bioaccumulation.

For example, As, Cr, Hg, Pb, and Cd concentrations must be under 10, 50, 1, 10, and 3 µg·L^−1^, respectively [1]. Such low values need to be determined by means of highly sensitive techniques, such as flameless atomic adsorption spectrophotometry (FAAS) [2], inductively coupled plasma optical emission spectroscopy (ICP-OES) [3], and inductively coupled plasma mass spectrometry (ICP-MS) [4], among others; however, these require expensive equipment and specialized technicians. These facts increase both the analysis time and the operation costs.

In order to avoid those inconveniences, alternative methodologies can be used for the quantification of HMI, such as electrochemical voltammetry. This technique offers, among other advantages, low-cost equipment, easy handling, and relatively fast analysis; in addition, it is suitable to be used as a portable quantification device [5,6,7,8,9]. Thus, regarding the determination of HMI, the improvement of the electrochemical performance and sensitivity to HMI are required. 

Analysts have invested major effort into the design of new electrochemical sensors, by taking advantage of the electrocatalytic effect of nanomaterials. This is aimed by the incorporation of metallic nanoparticles (MNPs) [10], oxide nanoparticles (ONPs), or carbon nanoallotropes (as carbon nanotubes or graphene) in the electrode components. These nanomaterials reduce the electron transfer resistance at the electrode surface, which leads to the decrease of the electron transfer limited process, and consequently the response of the electrode at low analyte concentration is catalyzed [11,12,13]. For example, it has been reported that with these modifications, the HMI concentration can be determined at values lower than 1 µg·L^−1^, which meets the WHO requirements for heavy metal quantification in drinking water samples.

More specifically, the incorporation of nanoparticles (NPs) on the sensor surface has shown good results for the determination of As [14,15,16], Cr [17], Cu [10,18,19], Pb [10,18,19,20], Cd [5], and Hg [7,21,22]. Most of these works used a screen-printed electrode (SPE) modified with different types of NPs (Pt-NPs, Ag-NPs or Au-NPs) as electrocatalysts. The main advantages of using SPEs are their versatility, and the fact that they can be used as one-use disposable sensors (avoiding any possible carryover contamination from previous measurements). Additionally, they are cost-efficient and easily tunable, as well as suitable to be incorporated in portable devices. 

There are different approaches to the modification of electrochemical sensors with nanomaterials. For instance, the modification used by Sanllorente-Méndez et al. [15] and Domínguez-Renedo et al. [17] was based on the electrochemical reduction of a PtCl_6_^2−^ solution, by applying two different potentials (+0.5 V for 0.01 s and −0.7 V for 10 s) to prepare Pt-NPs directly on the SPE. The main disadvantages of these procedures are that the distribution of the NPs along the electrode surface cannot be controlled, and the agglomeration of NPs is very common, directly influencing the electrochemical response of the sensor. 

On the other hand, Li et al. [7] carried out a dip coating strategy by immersing the electrode into an Au-NP colloidal solution overnight, after which the authors dried the electrode at 80 °C. A similar process was used by Pérez-Ràfols et al. [10] as they drop-casted Ag-NPs on the SPE surface, with a later drying stage of 30 min at 50 °C. In this case, the particle size distribution and morphology could be regulated by the NPs preparation; in addition, while this is not a time-consuming approach, there is still a lack control of the final distribution of the NPs on the modified surface. One alternative physical strategy used to customize surfaces is achieved by spin-coating (SC). SC is a common technique used to produce uniform thin films (e.g., of organic materials) with customized thicknesses on the order of micrometers and nanometers. For the case of the preparation of NP films, the coating depends on the NP concentration, and therefore SC could be a suitable method to modify screen-printed electrodes, although it requires specific instrumentation. 

Muñoz et al. [23] carried out the incorporation of NPs into carbon nanotubes/epoxy nanocomposite electrodes by physical and chemical approaches. These included the in situ modification of the transducer material (with control of NP spatial distribution: the physical mixing of NPs into the electrode matrix and drop attachment (drop-casting) of a NP-containing solutions. The overall results showed an enhancement of the electrochemical response to hydrogen peroxide as an analyte. Nevertheless, it is worth highlighting the importance of the physical and chemical stability of the substrate being modified by this in situ approach, due to the use of strong reducing agents (like sodium borohydride) that could affect the substrate. 

In this sense, the aim of this work is to evaluate the feasibility of different strategies to modify commercial, screen-printed, carbon nanofiber electrodes (SPCNFEs) with shaped silver nanoparticles, and therefore compare the electrocatalytic effect of these NPs on the determination of Cd(II) and Pb(II) in water samples. Ag-NPs were selected over Pt-NPs and Au-NPs, because they represent a less expensive alternative nanoelectrocatalyst, with tunable morphology and further feasible functionalization by simple approaches. All of this makes the electrochemical behavior of Ag-NPs easily customizable [24]. Specifically, three procedures of Ag-NP deposition are considered: drop-casting (DC), in situ (IS), and spin-coating (SC). In addition, the characterization of the NPs and the SPCNFE surface by means of electron microscopy is described. Finally, the electrochemical characterization by differential pulse anodic stripping voltammetry (DPASV) of the modified sensors is also presented.

## 2. Materials and Methods

### 2.1. Reagents and Solutions

All chemicals were analytical grade, and were used with no additional purification. Different reagents used for the Ag-NP synthesis (sodium citrate, sodium polystyrene sulfonic acid (SPSS), and silver nitrate) were supplied by Sigma-Aldrich (Munich, Germany), sodium borohydride by Panreac (Barcelona, Spain), and ascorbic acid by Scharlab (Barcelona, Spain). Lead(II) nitrate and cadmium(II) nitrate were purchased from Fluka (Buchs, Switzerland) and VWR International LTD (Radnor, PA, USA), respectively. Totals of 2072 µg·L^−1^ of Pb(II) and a 1124 µg·L^−1^ of Cd(II) solutions (corresponding to 10^−5^ mol·L^−1^) were prepared by sequential dilution from a 1000 mg·L^−1^ stock solution. Metal solutions were standardized by ICP-OES, with a Perkin Elmer model Optima 3200 (Waltham, MA, United States), or by ICP-MS, with an Agilent model 7500cx (Santa Clara, CA, United States). A 0.1 mol·L^−1^ acetate buffer solution (pH 4.5), prepared from acetic acid (Merck, Munich, Germany) and sodium acetate (Panreac, Barcelona, Spain), was used as electrolyte for constant pH, and to avoid the formation of metal hydroxocomplexes. All solutions were made with ultrapure water (18.2 MΩ·cm) obtained from a Milli-Q plus 185 system (Millipore, Burlington, MA, USA).

### 2.2. Electrodes

Commercial SPCNFEs (Dropsens, ref. 110CNF, Llanera, Spain), including working, counter, and reference electrodes, were used. The working electrode (WE) consisted on a disk of 4 mm diameter where the carbon nanofiber surface was modified with the silver nanoparticles (Ag-NP–SPCNFE).

### 2.3. Nanoparticle Synthesis

Ag nanoseeds (Ag-NS) and Ag nanoprisms (Ag-NPr) were prepared as follows, based on the methodology described elsewhere [25,26].

#### 2.3.1. Preparation of Ag Nanoseeds

Ag-NS were obtained by mixing under continuous stirring, with 5 mL of 2.5 mmol·L^−1^ sodium citrate, 0.25 mL of 500 mg·L^−1^ poly(styrenesulfonate) (PSS), and 0.3 mL of 10 mmol·L^−1^ sodium borohydride. Afterwards, a solution of 0.5 mmol·L^−1^ silver nitrate was continuously added to the previous solution at 2 mL·min^−1^ rate, by using a syringe pump from Kd Scientific, model KDS 510 (Holliston, MA, USA).

#### 2.3.2. Preparation of Ag Nanoprisms

Several aliquots in the range 400–1600 µL of the Ag-NS previously obtained were mixed with 5 mL of Milli-Q water and 75 µL of 10 mmol·L^−1^ ascorbic acid. Then, 3 ml of 0.5 mmol·L^−1^ silver nitrate were continuously added to each aliquot at 1 mL·min^−1^. Finally, 0.5 mL of 25 mmol·L^−1^ sodium citrate was added to each solution, in order to stabilize the obtained Ag-NPr.

### 2.4. Electrode Modification

#### 2.4.1. Drop-Casting Methodology

The same methodology and conditions as previously studied by Pérez-Ràfols [10] were used in the modification of the SPCNFEs by both Ag-NS and Ag-NPr. The deposition methodology consisted in placing 40 µL of the Ag-NPs in aqueous solution on the working electrode surface of the SPCNFE, and evaporating the solvent by heating it at 50 °C for 30 min. This procedure avoids damage to the Ag-NPs or electrical parts of the electrode, and at the same time, it ensures the removal of water. 

#### 2.4.2. Spin-Coating Methodology

Ag-NS and Ag-NPr were also used in the SPCNFE modification by the spin-coating methodology. A WS-650-8B spin coater from Laurell Technologies Corporation (North Wales, PA, United States) was used. The SPCNFE was attached to the center part of the spin coater by a vacuum system. Then, the different Ag-NPs were added onto the center part of the working electrode, by placing 20 µL of the colloidal solution. The SPCNFE was then spin-coated under a nitrogen atmosphere at 2000 rpm for 3 min. A second 20 µL drop of Ag-NP was placed onto the same place, and the same spin coating methodology was performed again on the electrode. The total volume was set to 40 µL, to be equal to that used in the drop-casting strategy, and coating conditions (time and speed) were optimized regarding film preparation studies [27].

#### 2.4.3. In situ Nanoparticle Synthesis on the Electrode Surface

In this methodology, a bare SPCNFE electrode was dipped in different beakers containing the following solutions: first, it was immersed in 3 mol·L^−1^ nitric acid for 1 h; then in 1 mol·L^−1^ sodium chloride; then in a 0.1 mol·L^−1^ silver nitrate; and finally, in 0.2 mol·L^−1^ freshly prepared sodium borohydride, each for 30 min [23]. 

### 2.5. Characterization of the Ag-Nanoparticles and of the Screen-Printed Carbon Nanofiber Electrode Surface

#### 2.5.1. UV/VIS Spectroscopy

The UV/VIS spectra showing the surface plasmon resonance (SPR) of the Ag-NS and Ag-NPr colloidal solutions were recorded by using an Agilent-Hewlett Packard spectrophotometer, model 8453 (Waldbronn, Germany).

#### 2.5.2. Scanning Electron Microscopy (SEM)

Colloid solutions of Ag-NS and Ag-NPr nanoparticles were characterized by using a Gemini scanning electron microscope from ZEISS® (Jena, Germany). Ag-NP samples were prepared as in a previous work [28]. In addition, the surface of the SPCNFE electrodes was also studied by the scanning electron microscope before and after the Ag-NP deposition. This aimed to determine the presence of NPs, as well as their spatial distribution on the SPCNFE with regard to the modification strategy: drop-casting, spin-coating, or in situ.

#### 2.5.3. Transmission Electron Microscopy (TEM)

For further characterization of the Ag-NPs synthetized in this work, a JEM-2010 transmission electron microscope from JOUL (Tokyo, Japan) was used. Ag-NP samples were also conditioned as in a previous work [28]. From the TEM images, the size distribution of the obtained Ag-NPs was determined, and the size distribution histograms were calculated as before [28], using the Image-J version 1.51m software.

### 2.6. Electrochemical Characterization of Ag-Nanoparticle–Screen-Printed Carbon Nanofiber Electrodes

Voltammetric studies of the SPCNFE modified electrodes were performed with a Multi Autolab/M204 Modular Multi Potentiostat/Galvanostat, as well as a personal computer with NOVA 2.1 software package to control the potentiostat and for the required data treatment, all from Metrohm (Herisau, Switzerland). 

Cyclic voltammograms of the bare and NP-modified electrodes were obtained in acetic acid/acetate buffer solution by scanning the potential from −1.00 to +1.00 V, at a scan rate of 0.01 V·s^−1^ and a step potential of 0.00244 V.

Differential pulse anodic stripping voltammetry (DPASV) measurements, using Ag-NP–SPCNFE for the determination of Pb(II) and Cd(II) ions, were performed. A deposition potential (*E_d_*) of –1.40 V, applied under stirring conditions during a deposition time (*t_d_*) of 180 s followed by a rest period (t_r_) of 5 s, were used. DPASV measurements were carried out under the following conditions: a scanning potential range from −1.40 to 0.00 V, a step potential of 5 mV, a pulse time of 50 ms, and a pulse amplitude of 50 mV. All experiments were performed at room temperature (22 ± 1 °C) and without oxygen removal.

Measurements of Pb(II) and Cd(II) ions by DPASV were performed using bare electrodes, and for each Ag-NP–SPCNFE prepared by the different modification methodologies. Initially, the electrodes were calibrated for each HMI. For this purpose, increasing concentrations of Pb(II) and Cd(II) solutions were added to an initial 40 mL of acetic/acetate buffer solution. Calibration samples ranged from 1.0 to 100.0, and from 1.0 to 75.0 µg·L^−1^ for Pb(II) and Cd(II), respectively.

## 3. Results and Discussion

### 3.1. UV/VIS Spectroscopy Characterization

**Figure 1:**
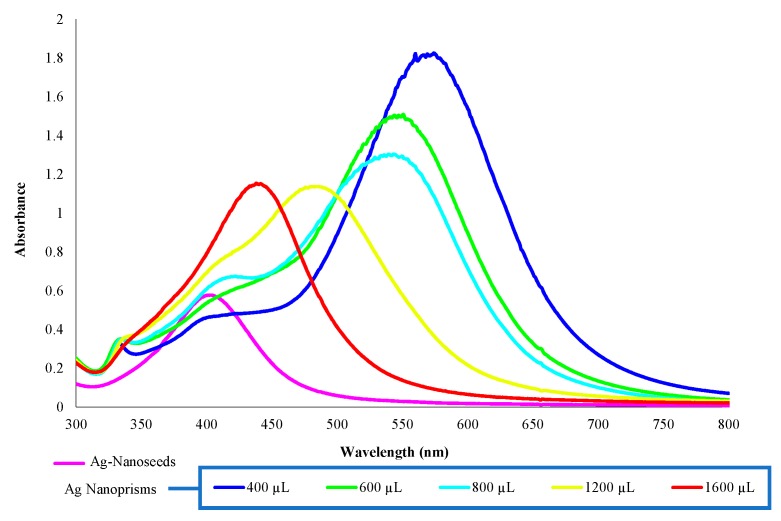
UV/VIS spectra obtained for the Ag-nanoseeds and Ag-nanoprisms synthesized by using different volumes of Ag-seed solution: 400, 600, 800, 1200, and 1600 µL.

The formation of the Ag-nanoparticles, perceived by simple visual observation of the color, was monitored by the UV-VIS spectra of the different colloidal solutions. Thus, to the initial Ag-NS suspension, different volumes ranging between 400 and 1600 µL of the Ag-NS colloidal solution were added. Figure 1 shows the spectra obtained for the Ag-NS and Ag-NPr synthesized by using the different volumes of Ag-seed solution already mentioned. It can be observed that the Ag-NPr nanoparticles with less seed solution (400 µL) showed an absorbance peak at 570 nm further from the initial seeds’ absorbance peak at 405 nm. However, colloidal solutions with higher amounts of the added Ag-NS solution presented absorbance bands that shifted to lower wavelengths, getting closer to the original Ag-NS solution (see Figure 1). The obtained wavelengths agreed with previously reported values, where silver colloids exhibited maximum absorbance within the range 400–500 nm, due to surface plasmon resonance (SPR) [29,30,31]. It must be mentioned that the peak absorbance of the 400 µL Ag-NS solution was higher than all the others, and when higher volumes of Ag-NS solution were added, peaks heights decreased to values getting closer to those presented by the initial Ag-NS solution. Moreover, the shift (from 405 nm in the initial Ag-NS solution) towards larger wavelengths (570 nm in 400 µL solution) could also indicate an increase in the mean diameter of Ag-nanoparticles [32,33]. For all this, the Ag-NS and the Ag-NPr obtained from a volume of 400 µL Ag-NS solution were used in the next studies.

### 3.2. Electron Microscopy Characterization

#### 3.2.1. Characterization of Ag-Nanoparticles by Transmission Electron Microscopy and Scanning Electron Microscopy

**Figure 2:**
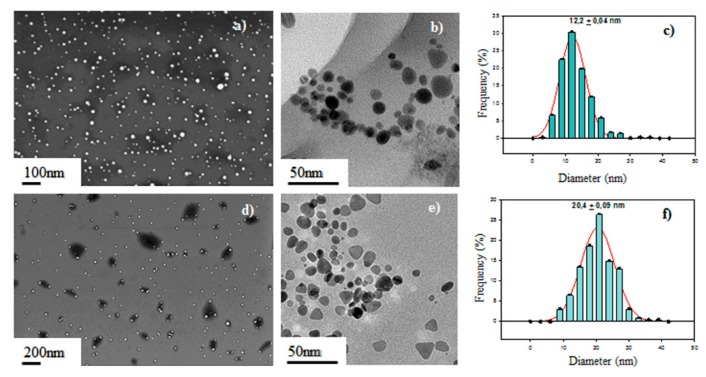
Characterization of the Ag-nanoseeds (**a**, **b**, **c**) and Ag-nanoprisms (**d**, **e**, **f**): scanning electron microscopy (SEM) images (**a**, **d**), transmission electron microscopy (TEM) images (**b**, **e**), and size distribution histograms (**c**, **f**).

Figure 2 shows the electron microscopy characterization of the studied Ag-NP samples. The SEM image of the Ag-NS can be observed in Figure 2a (white dots), confirming the effectiveness of the synthesis procedure followed. Additionally, Figure 2c shows the size distribution histogram obtained from a total of 400 Ag-NS. The nanoparticle counting was performed following the same procedure and using the same software as reported previously [28]. These results show that the Ag-NS obtained presented an average diameter of 12.2 ± 0.4 nm. 

From the TEM image presented in Figure 2b, it can be deduced that most of Ag-NS present a spherical shape. These structures are in good agreement with reported shapes of Ag-NPs [25,26].

The resulting SEM image of the Ag-NPr obtained can be observed as white dots in Figure 2d. The diameter distribution histogram of the Ag-NPr (see Figure 2f) show that the average particle size was 20.4 ± 0.09 nm. Compared with the Ag-NS, Ag-NPr are bigger and present a slightly wider size distribution than the Ag-NS. These results confirm the transformation of Ag-NS to Ag-NPr, as described previously [25,26].

#### 3.2.2. Electrode Characterization by Scanning Electron Microscopy

**Figure 3:**
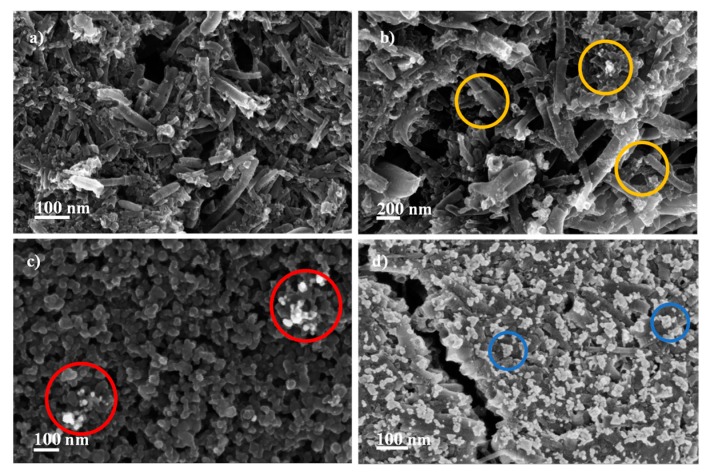
SEM InLens images of the electrodes used in this work. (**a**) Bare, commercial, screen-printed carbon nanofiber electrode (SPCNFE); (**b**) Ag-NS –SPCNFE modified by drop-casting method; (**c**) Ag-NS-SPCNFE modified by the in situ synthetic method; (**d**) Ag-NS–SPCNFE, modified by the spin-coating method. Examples of Ag-nanoparticle location is indicated with colored circles.

In Figure 3, the SEM InLens images of the different electrodes obtained as explained above is presented, in order to compare the final surfaces obtained. The Ag-NS were located in the carbon fibers, identified as circled white spots in the images of Figure 3b–d. 

Figure 3c shows the Ag-NP incorporation by the in situ methodology. It can be observed that the modification of the electrode surface was not acceptable, due the low amount of Ag-NS deposited. In addition, the mechanical resistance of the SPCNFE was compromised, as some changes on the substrate were observed (change of color of the electrode connectors) that could be caused by the NaBH_4_. Additionally, blank electrodes were prepared by the IS approach, and their response signal was lost in all cases, showing that this modification strategy was not appropriate to functionalize the electrode surface with NPs. Therefore, the IS strategy was not used for the determination of heavy metals. 

The SEM images of the electrodes prepared by the drop-casting of Ag-NS (Figure 3b) indicate, in this case, the modification of the electrode, with Ag-NS visible over the electrode surface. Finally, the electrodes prepared by the spin-coating approach showed the surface modification of the SPCNFE (Figure 3d) with a larger number of deposited Ag-NPs, as compared with the two previous methodologies. This effect could be attributed to a more uniform distribution of the nanoparticle suspension over the electrode substrate, due to the speed of the spin-coating process itself. Based on the characterizations performed, it was decided to continue the work only using the two electrodes that showed successful incorporation of the NPs to the electrode surface. 

### 3.3. Electrochemical Characterization of the Electrodes

#### 3.3.1. Preliminary Studies of the SPCNFE Modification with Ag-Nanoparticles

In order to determine if the modification of SPCNFEs with Ag-nanoparticles resulted in the enhancement of their electrochemical response, cyclic voltammetry (CV) and differential pulse anodic stripping voltammetry (DPASV) were performed. 

**Figure 4:**
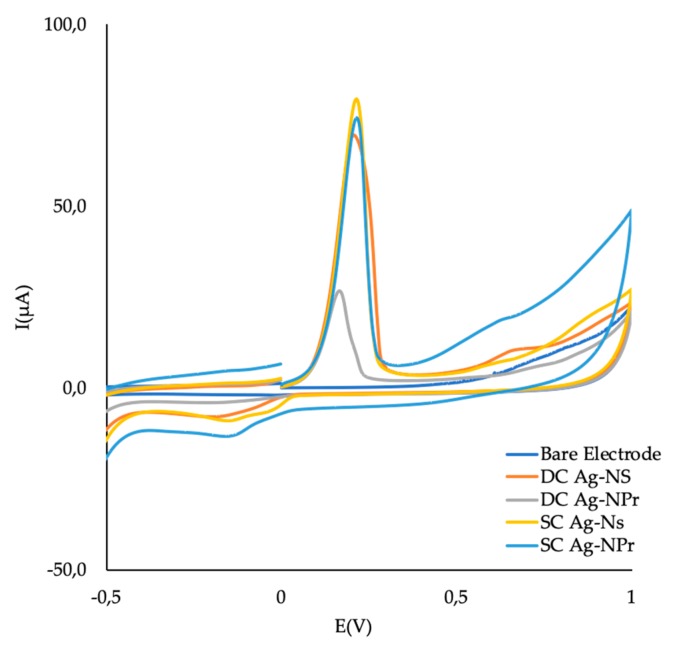
Cyclic voltammograms in acetic acid/acetate buffer at pH 4.5 for Ag-nanoseed- and Ag-nanoprism-based SPCNFE electrodes, obtained by using either drop-casting (DC) or spin-coating (SC) methodologies.

First, the cyclic voltammograms of both the bare and the Ag-NP-modified electrodes were carried out, as seen in Figure 4. It can be seen that the current intensity of the oxidation peaks at 0.2 V (Ag-NPs) of the Ag-NS–SPCNFE and Ag-NPr–SPCNFE are very similar, suggesting that the Ag-NP concentration on the WE surface would be comparable, which is important for the evaluation of both strategies. 

It seems that SC strategy offers a more consistent approach in terms of amount of NPs incorporated to the SPCNFE. DC strategy depends on factors like solvent evaporation and appropriate location of the drop; this can explain the different signal obtained regarding the number of NPs incorporated, although for the case of DC for Ag-NPr, the signals agree accordingly with the SC strategy. 

As seen in Figure 4, current peaks obtained in the case of Ag-NS–SPCNFE were higher than in the case of Ag-NPr–SPCNFE. Even though this effect might be caused by the fact that the amount of Ag-NPs on both electrode surface could be different, due to the different Ag-NP concentrations in the colloidal solutions, another reason could be related to the specific surface area of the Ag-NPs. As was shown in Figure 2, smaller nanoparticles were obtained in the case of Ag-NS than in Ag-NPr, and this would probably result in an enhanced response of the Ag-NS-SPCNFE. Nanoparticles exhibit a higher reactive surface, due to the increase of a high number of surface atoms to volume ratio, which leads to a high density of active sites. These features create NPs with different shapes and sizes, preferential reactivity, and selectivity [34].

**Figure 5:**
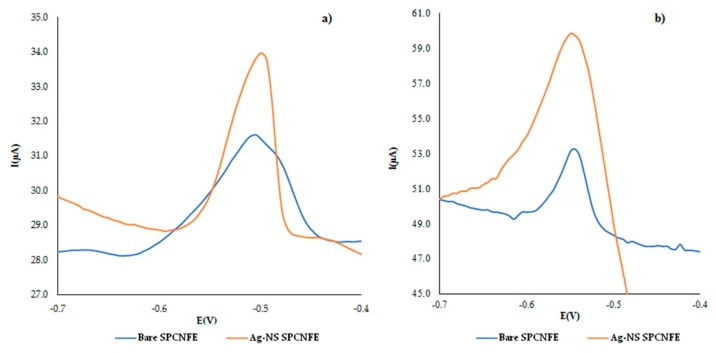
Differential pulse anodic stripping voltammetry (DPASV) measurements of non-modified SPCNFE and Ag-nanoseed–SPCNFE electrodes, obtained for 70 µg·L^−1^ of (**a**) lead(II) or (**b**) cadmium(II) ions. Experimental conditions: acetic acid/acetate buffer pH 4.5, with an *E_d_* of −1.40 V applied during a *t_d_* of 180 s, and employing a step potential of 5 mV, a pulse time of 50 ms, and a pulse amplitude of 50 mV.

In order to study the electrochemical response, comparing a bare SPCNFE electrode and an Ag-NP–SPCNFE in the determination of Pb(II) or Cd(II), differential pulse anodic stripping voltammetry (DPASV) measurements were performed in solutions containing either 70.0 µg·L^−1^ of Pb(II) or Cd(II). From the results obtained (see Figure 5), it can be concluded that the modification of the electrodes by Ag-NPs caused a significant increase in the electrode response, and would be an interesting alternative in the determination of both metal ions.

#### 3.3.2. Study of Ag-NS–SPCNFE Electrodes Obtained by Either Drop-casting (DC) or Spin-Coating (SC) Methodologies

Single calibration curves by DPASV were performed by increasing the concentration of Pb(II) and Cd(II) in the ranges from 1.0 to 100.0 and from 1.0 to 75.0 µg·L^−1^, respectively. The same procedure was followed for either DC or SC electrodes. From the data obtained, detection limits were determined by using the Miller and Miller procedure [35,36].

**Figure 6:**
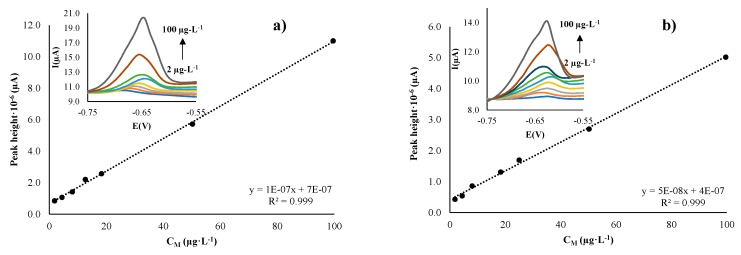
Calibration curves and DPASV measurements (insets) for Pb(II) calibration, obtained with the Ag-nanoseed–SPCNFE electrodes using (**a**) drop-casting and (**b**) spin-coating methodologies. Same experimental conditions as in Figure 4.

Results of the calibration parameters as limits of detection (LODs), linear ranges, and linearity are listed in Table 1. The limit of quantification (LOQ) is considered as the lower value of the linear range. As an example, the voltammogram related to the Pb(II) response and the corresponding calibration plot of Ag-NS–SPCNFE for DC and SC deposition methods are shown in Figure 6. 

It can be seen from the DPASV curves presented in Figure 6 that a stable oxidizing peak appears around −0.64 V, which corresponds to the increasing concentration of Pb(II) in both approaches. Comparing the results obtained with Ag-NS with the ones obtained with the bare electrode, it can be concluded that the electrode electrocatalytic response is clearly enhanced. This result agrees with previous reported works that stated the effect of Ag-NPs on the increased sensitivity and analytical features of modified SPCNFEs [10,28].

Table 1 shows that, in all cases, electrodes presented good and similar performance in terms of LODs, with slightly better values in the case of the spin-coating method. In terms of metal ion response, it can be observed that Ag-NS electrodes showed better linear regressions and wider linear ranges in the Pb(II) calibrations than in the Cd(II) response. This behavior was obtained for both modification strategies, and it seems that the electrocatalytic enhancement relays only on the presence of the NPs, but does not seem to be associated with the incorporation route. As was seen in the SEM images, in SC deposition most of the NPs are homogenously distributed on the surface of the electrode (externally localized), while for DC, it seems that there is some diffusion into the matrix (internally localized). One crucial parameter to control in SC is the spin acceleration, which drives the fluid thinning, solvent evaporation, and consequently, the film formation [27]. On the other hand, the DC deposition depends on the evaporation rate, due to the temperature and the uniform coating during the casting. It seems that the interaction between the casting and evaporation in DC leads to NP penetration into the nanofiber matrix.

In all cases, the LOD values obtained for both deposition methods are close to 3 µg·L^−1^. It is important to point out that these values are below or at least at the same order of magnitude of the legislated values, in the case of Pb(II) and Cd(II) concentrations in drinking waters [1]. 

#### 3.3.3. Study of Ag-NPr–SPCNFEs, Prepared by Either Drop-casting (DC) or Spin-coating (SC) Methodologies

**Figure 7:**
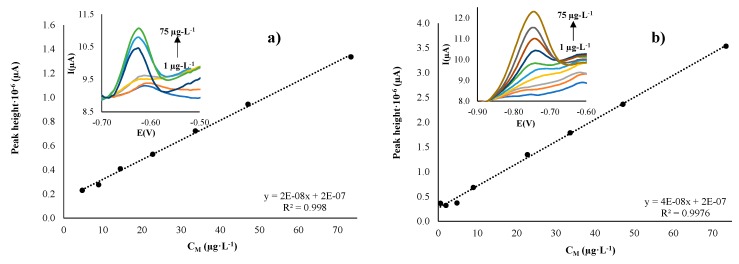
Calibration curves and DPASV measurements (insets) for Cd(II) calibration, obtained with the Ag-nanoprism–SPCNFE electrodes, using (**a**) drop-casting and (**b**) spin-coating methodologies. Same experimental conditions as in Figure 4.

By following a similar procedure as before, Ag-NPr electrodes were studied, and their response to Pb(II) and Cd(II) concentrations was determined. The results of the calibration parameters are presented in Table 1. From this data, it can be determined that slightly better electrochemical performance was observed in the case of the Cd(II) response for this kind of Ag-NP. In Figure 7, a stable and measurable signal can be observed when Cd(II) concentration is increased. This performance was seen in both DC and SC methodologies, and the current signal improved in a relevant way compared with the bare electrode. 

From the data presented in Table 1, it can be pointed out that Ag-NPr electrodes presented slightly better LOD values, better linear regressions, and wider linear ranges in the Cd(II) calibrations than in the Pb(II) response. However, non-significant differences were seen in this case, in terms of drop-casting or spin-coating methodologies. This would confirm the results mentioned for Ag-NS-SPCNFEs, that the modification methodology would not be a determinant factor in the enhancement of the response of the electrodes. 

Additionally, as it was already mentioned, LODs obtained for Ag-NPr–SPCNFEs in both deposition methods and for both studied ions were around 3 µg·L^−1^. These values are also below the legislated values for the determination of Pb(II) and Cd(II) concentration in drinking waters. Nevertheless, a comparison between all the response characteristics for both Ag-NPs studied could conclude that Ag-NS would be more suitable in the determination of Pb(II); meanwhile, Ag-NPr would be more appropriate for determining Cd(II). This can be mainly attributed to the different reactivity that these NPs can have, due to their shape and size difference. The shape and size of a nanoparticle directly influence the disposition of exposed atoms on its surface, making it have more electrocatalytically active sites (edge and corner sites) [37,38]. These active sites give preferential catalytic activity, as has been shown in studies concerning the electroreduction of CO_2_ to CO. Regarding the preferential electrocatalytic effect for the model ions studied, further studies are required.

## 4. Conclusions

In this work, three different deposition methodologies in situ, drop-casting, and spin-coating have been evaluated as feasible strategies for the modification of screen-printed carbon nanofiber electrodes (SPCNFEs), with two differently shaped nanoparticles: Ag-NS and Ag-NPr. For this, the formation of each type of Ag-NP was monitored by UV-VIS. Moreover, electron microscopy was used in the characterization of their size, shape, and distribution on the SPCNFE surface. The obtained Ag-NS presented an average diameter of 12.2 ± 0.04 nm, and in general showed a spherical shape. On the other hand, in the case of Ag-NPr, the obtained average particle size was 20.4 ± 0.09 nm, and among a variety of shapes, mostly showed a triangular shape. The electrode modification approaches were studied by scanning electron microscopy. In two of them, drop-casting and spin-coating, SEM images indicated a correct modification of the electrode, with Ag-NPs embedded inside or all over the carbon nanofibers. Additionally, in the case of the spin-coating methodology, SEM images showed a strong modification of the SPCNFEs, with many and more uniform Ag-nanoparticles deposited, due to the high speed of the spin coater. Finally, the in situ methodology showed a low number of Ag-NS deposited on the electrode surface, as well as a deep alteration of this surface. Those facts suggest that the in situ synthesis methodology was not appropriate for the electrode modification, and it was discarded.

Finally, electrochemical characterizations of the Ag-NP–SPCNFEs and their application in the determination of Pb(II) and Cd(II) ions were carried out. The results obtained in the case of Ag-NS- and Ag-NPr based SPCNFEs for both studied metal ions, as well as for the DC and SC deposition strategies, indicated appropriate and similar performances in terms of LOD, with values around 3 µg·L^−1^. In terms of metal ion response, it was observed that Ag-NS electrodes showed better linear regressions and wider linear ranges in the case of Pb(II) calibrations, while for Ag-NPr electrodes better results were obtained for the Cd(II) response. This behavior was obtained for both DC and SC methodologies. A comparison between all the response characteristics for both Ag-NPs studied could conclude that Ag-NS would be more suitable in the determination of Pb(II), while Ag-NPr would be more appropriate in determining Cd(II). On the other hand, an evaluation of the effect of the modification methodology on the Ag-NP–SPCNFE response indicates that using DC or SC deposition methods seem not to be a parameter that enhances the response of the SPCNFEs, even though better Ag-NP distribution was observed by SEM images in the case of SC methodology. Therefore, it can be concluded that the spatial distribution of the NPs in the SPCNFEs does not play a significant effect on their detection, under the conditions of the present study. However, their presence is the one giving the electrocatalytic enhancement, so the electrode substrate acts as the only component during the electrochemical detection. Nevertheless, the SC approach can be considered more time- and cost-efficient, which makes it a simpler approach for the modification of electrodes. In addition, good reproducibility was obtained in the assays (RSD% < 6 %). Finally, LODs obtained in both deposition methods for Ag-NPs for both studied ions (around 3 µg·L^−1^) are below or at least the same order of the legislated values, in the case of Pb(II) and Cd(II) concentration in drinking water. For all this, Ag-NP–SPCNFEs could be an accurate, portable, and sensible analytical system for the determination of Pb(II) or Cd(II) in natural waters. 

## Figures and Tables

**Table 1:** Calibration parameters as limits of detection (LODs), linear ranges, and linearity obtained for the determination of Pb(II) and Cd(II) on Ag-nanoseed–SPCNFE and Ag-nanoprism–SPCNFE, as well as for drop-casting (DC) and spin-coating (SC) methodologies.

	Pb(II)			Cd(II)		
Deposition Ag-NPs	LOD (µg·L^−1^) (RSD%) *	Linear Range (µg·L^−1^)	R^2^	LOD (µg·L^−1^) (RSD%) *	Linear Range (µg·L^−1^)	R^2^
DC Ag-NS	3.3 (1.6)	10.9–99.6	0.9990	3.7 (2.1)	12.2–73.4	0.9923
SC Ag-NS	2.8 (1.3)	9.4–99.6	0.9990	2.4 (1.3)	8.1–73.4	0.9976
DC Ag-NPr	3.1(5.6)	10.3–18.3	0.9840	2.2 (1.3)	7.4–73.4	0.9980
SC Ag-NPr	3.4(3.0)	11.3–50.3	0.9911	2.1 (1.3)	6.9–73.4	0.9976

* Data in parenthesis represent the relative standard deviation (RSD %).
